# Long‐term follow‐up of the TRED‐HF trial: Implications for therapy in patients with dilated cardiomyopathy and heart failure remission

**DOI:** 10.1002/ejhf.3475

**Published:** 2024-09-30

**Authors:** Leanne Cheng, Daniel Hammersley, Aaraby Ragavan, Saad Javed, Srinjay Mukhopadhyay, John Gregson, Jennie Han, Zohya Khalique, Amrit Lota, Antonis Pantazis, A. John Baksi, Gerald Carr‐White, Antonio de Marvao, James Ware, Upasana Tayal, Dudley J. Pennell, John G.F. Cleland, Sanjay K. Prasad, Brian P. Halliday

**Affiliations:** ^1^ National Heart & Lung Institute Imperial College London UK; ^2^ Inherited Cardiovascular Conditions Care Group & Cardiovascular Magnetic Resonance Unit Royal Brompton and Harefield Hospitals, part of Guy's and St Thomas NHS Foundation Trust London UK; ^3^ London School of Hygiene and Tropical Medicine London UK; ^4^ Inherited Cardiovascular Conditions Group Guy's and St Thomas NHS Foundation Trust London UK; ^5^ British Heart Foundation Centre of Research Excellent, School of Cardiovascular and Metabolic Medicine and Sciences King's College London London UK; ^6^ MRC Laboratory of Medical Sciences Imperial College London UK; ^7^ School of Cardiovascular and Metabolic Health University of Glasgow Glasgow UK

**Keywords:** Dilated cardiomyopathy, Heart failure, Withdrawal, Medical therapy, Relapse, Remission

## Abstract

**Aims:**

In TRED‐HF, 40% of patients with recovered dilated cardiomyopathy (DCM) relapsed in the short term after therapy withdrawal. This follow‐up investigates the longer‐term effects of therapy withdrawal.

**Methods and results:**

TRED‐HF was a randomized trial investigating heart failure therapy withdrawal in patients with recovered DCM over 6 months. Those randomized to continue therapy subsequently withdrew treatment between 6 and 12 months. Participants were recommended to restart therapy post‐trial and were followed until May 2023. Clinical outcomes are reported in a non‐randomized fashion from enrolment and from the end of the trial. The primary outcome was relapse defined as ≥10% reduction in left ventricular ejection fraction to <50%, doubling in N‐terminal pro‐B‐type natriuretic peptide to >400 ng/L, or clinical features of heart failure. From enrolment to the last follow‐up (median 6 years, interquartile range 6–7), 33 of 51 patients (65%) relapsed. The 5‐year relapse rate from enrolment was 61% (95% confidence interval [CI] 45–73) and from the end of the trial was 39% (95% CI 19–54). Of 20 patients who relapsed during the trial, nine had a recurrent relapse during follow‐up. Thirteen relapsed for the first time after the trial; seven had restarted low intensity therapy, four had not restarted therapy and two did not have therapy withdrawn. The mean intensity of therapy was lower after the trial compared to enrolment (mean difference −6 [−8 to −4]; *p* < 0.001). One third of relapses during follow‐up had identifiable triggers (arrhythmia [*n* = 4], pregnancy [*n* = 1], hypertension [*n* = 1], infection [*n* = 1]). Corrected atrial fibrillation was associated with reduced risk of relapse (hazard ratio 0.33, 95% CI 0.12–0.96; *p* = 0.042).

**Conclusions:**

The risk of relapse in the 5 years following the TRED‐HF trial remained high. Restarting lower doses of heart failure medications at the end of the trial, external triggers and disease progression are likely to have contributed to relapse.

## Graphical abstract

Long‐term follow‐up (FU) of the TRED‐HF trial: persistent high‐risk of relapse following therapy withdrawal in recovered dilated cardiomyopathy (DCM), likely contributed to by low‐dose therapy and exposure to external triggers. LVEF, left ventricular ejection fraction.

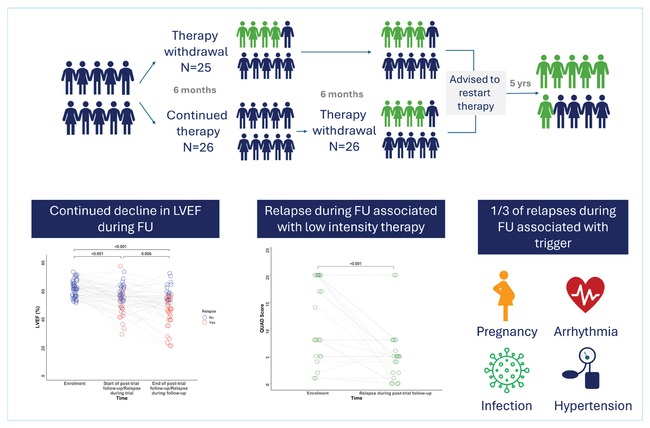

## Introduction

The incidence of left ventricular reverse remodelling and heart failure (HF) remission in patients with dilated cardiomyopathy (DCM) is increasing following advances in HF therapy.^1^, ^2^, ^3^ Contemporary studies suggest that left ventricular reverse remodeling may be observed in more than 50% by 12 months.^4^, ^5^ Increasing recognition of this phenomenon has prompted the definition of an additional HF phenotype, HF with improved ejection fraction (HFimpEF).^6^ Whilst it is well established that patients with HFimpEF have a better prognosis than those with persistently reduced left ventricular ejection fraction (LVEF), they are still at risk of adverse outcomes.^2^, ^3^, ^7^, ^8^ In the TRED‐HF (Therapy withdrawal in REcovered Dilated cardiomyopathy‐Heart Failure) trial,^9^ 40% of patients with a history of DCM, who had resolution of symptoms and normalization of LVEF and natriuretic peptides, relapsed within 6 months of withdrawing pharmacological therapy. These findings have helped guide healthcare professionals and patients to balance the risks and benefits in decisions regarding changes to guideline‐directed medical therapy amongst patients with the most complete reverse remodelling. However, there remains many questions. What is the effect of therapy withdrawal in the longer term? What are the predictors of future relapse in patients with DCM and HF remission and how can relapse be prevented? Do some patients have durable recovery without the need for ongoing medication? What medications are most important for maintaining HF remission?

We report the extended 5‐year follow‐up of patients enrolled in the TRED‐HF trial and describe the incidence of relapse in the long term and its predictors, including the intensity of medical therapy and precipitating events.

## Methods

### Study design and participants

TRED‐HF was an open‐label, pilot trial investigating the effects of withdrawal of medical therapy in patients with recovered DCM^9^ that randomized 51 patients (1:1) to 6 months of phased withdrawal or continuation of treatment, followed by a 6‐month single‐arm crossover phase.

Inclusion and exclusion criteria have been previously described.^9^ Patients aged >16 years were included if they had a previous diagnosis of DCM with a documented LVEF of ≤40% and were currently treated with at least one guideline‐directed medical therapy (an angiotensin‐converting enzyme inhibitor [ACEi], angiotensin receptor blocker [ARB], beta‐blocker [BB], mineralocorticoid receptor antagonist [MRA], or a loop diuretic). Recovery was defined as: (i) no symptoms of HF, (ii) a LVEF ≥50% and a left ventricular end‐diastolic volume indexed to body surface area (LVEDVi) within the normal range, (iii) and plasma N‐terminal pro‐B‐type natriuretic peptide (NT‐proBNP) concentration of <250 ng/L. Trial investigations were performed at a single centre (Royal Brompton and Harefield Hospitals, London, UK). The cause of DCM was adjudicated by an expert cardiologist.

All patients provided written informed consent. The trial was approved by the National Research Ethics Committee (16/LO/0065) and is registered on ClinicalTrials.gov (NCT02859311). The investigation conforms with the principles outlined in the Declaration of Helsinki.

### Outcomes and follow‐up

After completing the trial, participants continued routine clinical care with their usual physicians. All participants and their physicians were advised that HF therapy should be restarted, with ongoing imaging surveillance performed on an annual basis. All healthcare records including clinic letters, imaging reports, blood tests and summary care records were gathered from primary and secondary care sources. Patients were contacted to confirm current medication, the occurrence of events and to complete the Kansas City Cardiomyopathy Questionnaire (KCCQ‐12) at the end of post‐trial follow‐up (online supplementary *Figure S* *2*). The plan for medical therapy instituted by the clinical team at the first follow‐up was recorded as the initial post‐trial treatment. For example, if the patient was prescribed bisoprolol 2.5 mg/daily with the plan to up‐titrate to maximum tolerated dose, and 6 weeks later achieved a maximum dose of 7.5 mg/daily, 7.5 mg/daily was recorded as the initial post‐trial treatment. QUAD scores were calculated once medications had been re‐established after the first follow‐up and at the final post‐trial follow‐up.^10^ The QUAD score was developed to document the intensity of HF medical therapy.^10^ This scoring system uses a point‐based classification to reflect the prescription and dose of any renin–angiotensin system inhibitor (RASi) such as ACEi/ARB/angiotensin receptor–neprilysin inhibitor (ARNI), BB, MRA and sodium–glucose cotransporter 2 inhibitor (SGLT2i). Individuals are allocated a score for each medication prescribed. A score of 4 is allocated if they are on ≥50% of the target dose and a score of 1 is given if they are on <50% of the target dose. Patients taking all drug classes are given an extra 8 points. The maximum score is 24 and is considered optimal therapy. However, as SGLT2is were not introduced until 24 February 2021, 20 was taken as the maximum therapy before the date of introduction. Target doses were taken from the European Society of Cardiology guidelines (online supplementary *Table* *S1*).^11^

The primary endpoint was a relapse of DCM. This is reported (i) any time from enrolment to the end of follow‐up, and (ii) from the end of the trial to the end of follow‐up. During the trial, this was defined by at least one of the following: (i) a reduction in LVEF by ≥10% and to <50%, (ii) a 10% increase in LVEDV and to above the normal range, (iii) a two‐fold rise in NT‐proBNP concentration to >400 ng/L; (iv) or clinical evidence of HF, based on signs and symptoms determined by clinicians. The primary endpoint during the follow‐up period only considered (i), (iii), and (iv). The LVEDV criterion (ii) was not included as part of the primary endpoint in the post‐trial phase due to variations in imaging modality. For patients whose LVEF had improved to >50%, recurrent relapses were defined by recurrent deterioration in LVEF by ≥10% and to <50%. The lowest LVEF at each relapse was recorded. The imaging closest to the date of relapse was taken. Secondary endpoints included all‐cause mortality, unplanned cardiovascular hospitalizations, and change in LVEF and KCCQ‐12 scores. LVEF and KCCQ‐12 scores at the end of the trial were taken as the start of post‐trial follow‐up.

### Statistical analyses

Baseline characteristics by randomized groups were previously described^9^. Comparisons in *Table* *1* are made based on the occurrence of relapse at any point during or after the trial period using Mann–Whitney test for continuous variables or Fisher's exact test for categorical variables. Data are displayed as median and interquartile range (IQR) or number (%). The occurrence of the primary endpoint is displayed using Kaplan–Meier cumulative incidence curves, with the inclusion of 5‐year event rates. The total number of primary outcome criteria satisfied is represented via Venn diagrams. Quantitative secondary endpoints were compared between serial time points amongst all patients using paired *t*‐tests and graphically displayed using dot plots. To handle missing data, the main analyses used a complete‐case approach (i.e. only including patients with complete data at all time‐points). Sensitivity analyses used multiple imputation with chained questions and 100 imputed datasets. Potential predictors of relapse were explored using univariable Cox proportional hazards modelling. NT‐proBNP was log‐transformed to achieve a more normal distribution.

**Table 1:** Baseline characteristics of patients at enrolment

	Relapse (*n* = 33)	No relapse (*n* = 18)	*p*‐value
Age, years	56 (46–64)	52 (44–63)	0.330
Men	20 (61)	14 (78)	0.352
Time since initial DCM diagnosis, months	155 (75–257)	125 (52–228)	0.340
LVEF at initial diagnosis, %	25 (20–33)	27 (20–31)	0.972
Absolute improvement in LVEF, %	31 (25–39)	28 (23–36)	0.397
Time with LVEF >50% prior to trial, months	28 (8–45)	18 (6–44)	0.678
Previous unplanned HF admission	17 (52%)	15 (83%)	**0.015**
Past medical history			
Current or previous alcohol >14 units per week	12 (36)	5 (28)	0.757
Previous atrial fibrillation	4 (12)	8 (44)	**0.035**
Previous hypertension	2 (6)	2 (11)	0.607
Diabetes mellitus	1 (3)	0 (0)	1.000
Family history of DCM	5 (15)	1 (6)	0.405
Causal and other factors in DCM			
Adjudicated cause of DCM			
Idiopathic	22 (67)	13 (72)	0.495
Familial	6 (18)	1 (6%)	–
Environmental Insult	5 (15)	4 (22)	–
Truncating variant in TTN	9 (27)	2 (11)	0.288
Other variables			
Body surface area, m^2^	2.0 (1.7–2.2)	2.1 (1.9–2.3)	0.136
Left bundle branch block	6 (18)	1 (6)	0.398
QRS duration, ms	96 (86–109)	94 (88–105)	0.721
NT‐proBNP, ng/L	90 (44–153)	50 (33–83)	**0.014**

Data are presented in *n* (%) or median (interquartile range). Groups are stratified based on the occurrence of relapse at enrolment to the end of follow‐up. Measurements were made at baseline. Mann–Whitney test was used for continuous variables and Fisher's exact test was used for categorical variables.

DCM, dilated cardiomyopathy; HF, heart failure; LVEF, left ventricular ejection fraction; NT‐proBNP, N‐terminal pro‐B‐type natriuretic peptide.

A *p*‐value of < 0.05 was taken as significant and all hypothesis tests were two‐tailed. Statistical analyses were done with Prism9 (version 9.5.1), RStudio (version 4.2.2) and Stata version 18.

## Results

### Study population and patient characteristics

Extended post‐trial follow‐up data were obtained for all 51 patients. Median follow‐up time from the start of the trial was 6 (IQR 6–7) years. The median number of cardiac imaging studies performed was 3 (IQR 2–5). Two patients who did not otherwise fulfil criteria for relapse had no imaging performed after completing the trial. Both remained asymptomatic.

Characteristics at enrolment are displayed in *Table* *1* based on the occurrence of the primary endpoint at any stage from enrolment to the end of follow‐up. The median age at enrolment was 55 (IQR 45–64) years and 34 patients (67%) were men. Patients who relapsed were less likely to have a history of atrial fibrillation (AF) (12% vs. 44%, *p* = 0.035) and had higher plasma NT‐proBNP concentrations at enrolment (medians: 90 vs. 50 ng/L, *p* = 0.014).

### Primary outcome events and secondary causes

**Figure 1:**
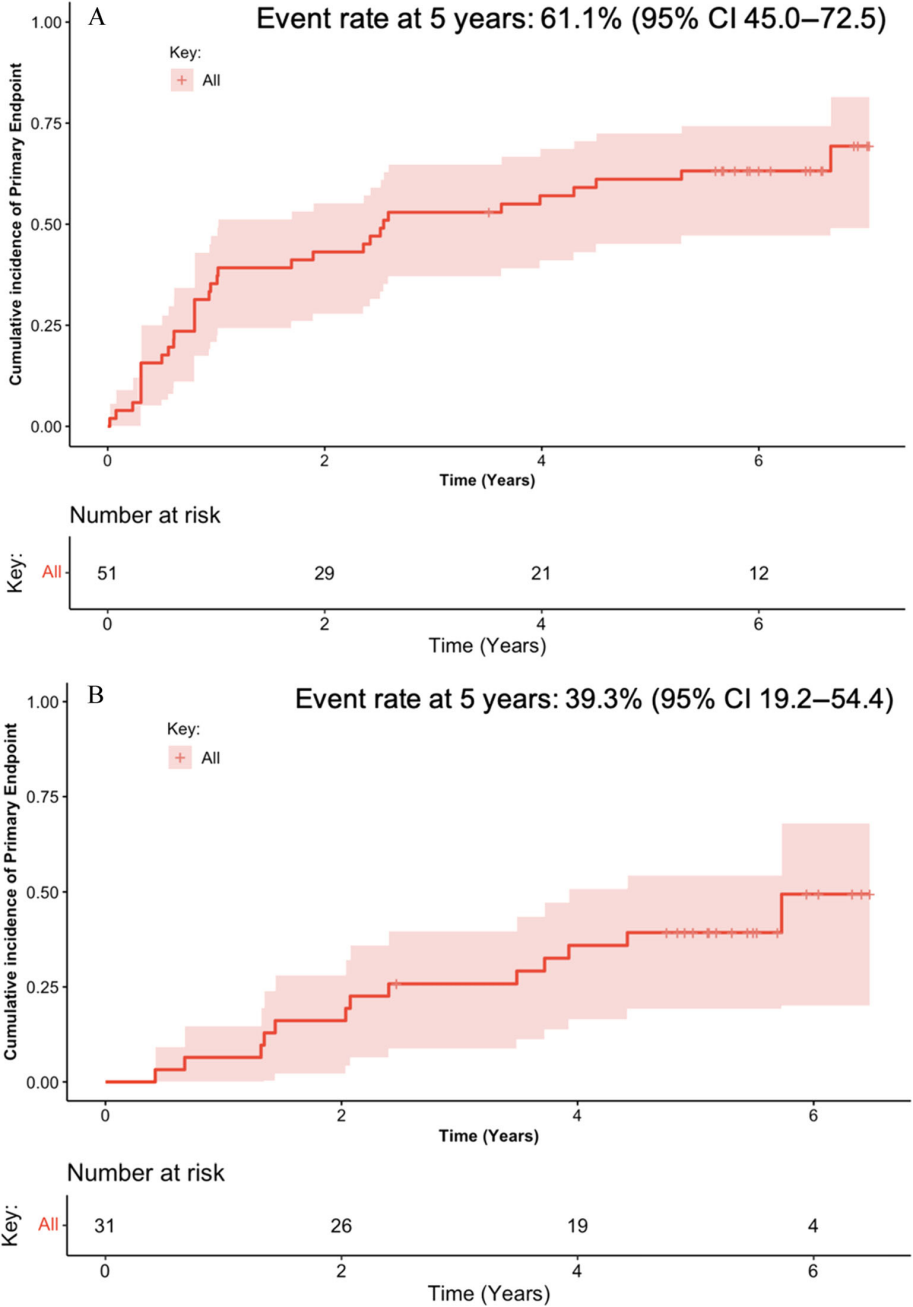
Cumulation of incidence curves of the primary endpoint (*A*) at any time from enrolment and (B) during the post‐trial follow‐up. Only 31 patients were compared in (*B*), excluding the 20 patients who relapsed during the trial. CI, confidence interval.

**Figure 2:**
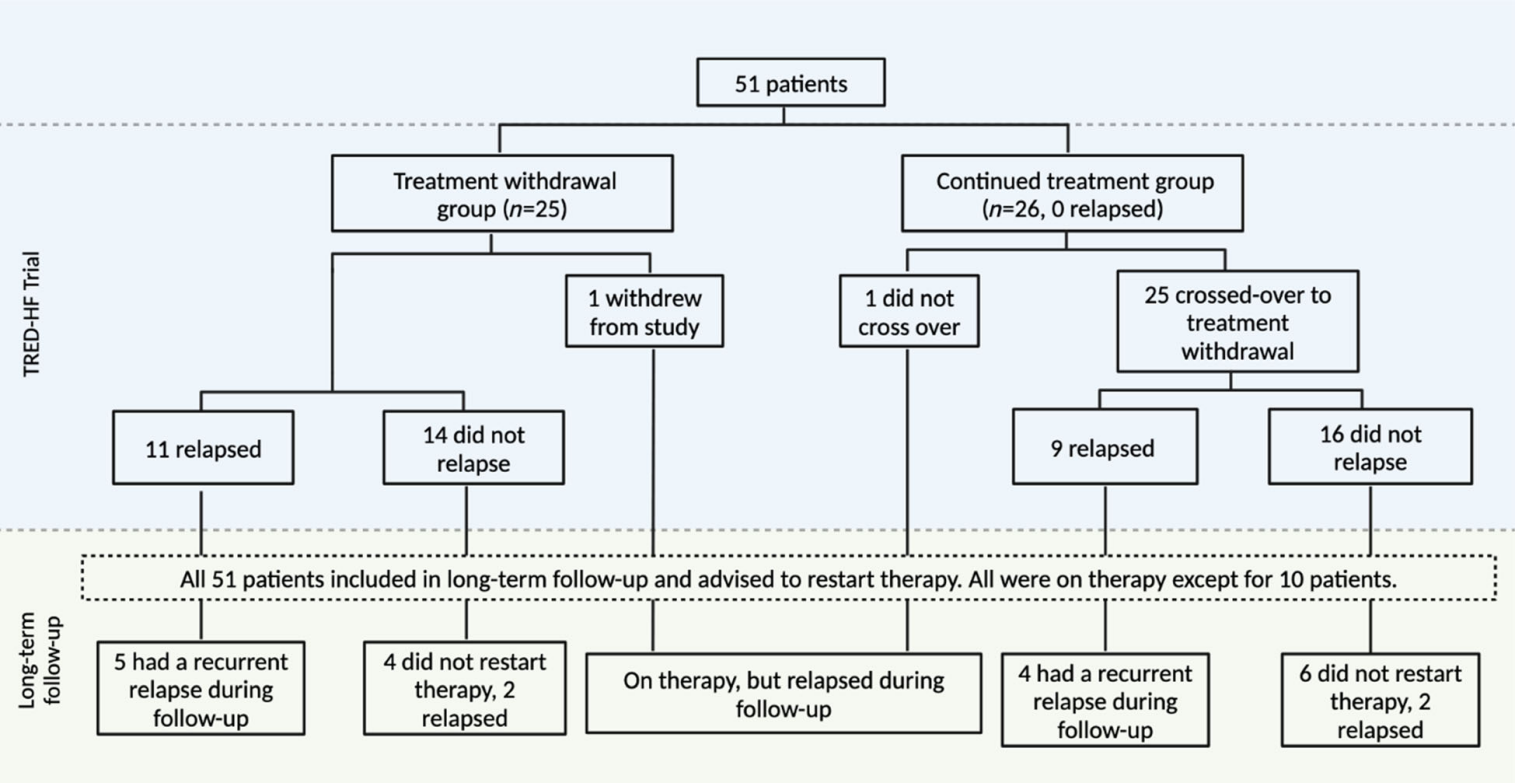
Follow‐up profile. One patient withdrew shortly after enrolment from the original trial and relapsed during the post‐trial follow‐up period. Follow‐up data were available for all 51 patients.

From enrolment to the end of follow‐up, 33 of 51 (65%) patients met the primary endpoint. The Kaplan–Meier estimate of the 5‐year event rate from enrolment was 61% (95% confidence interval [CI] 45–73) (*Figure* *1A*). The 5‐year event rate from the end of the trial was 39% (95% CI 19–54) (*Figure* *1B*). Of the 31 patients who did not relapse during the trial, 13 (42%) relapsed during the post‐trial follow‐up period. Nine of 20 (45%) patients who relapsed during the trial subsequently had recurrent relapses; eight had one recurrent relapse, while one patient had two. In total, 22 (43%) patients relapsed in the post‐trial follow‐up period. Amongst the 41 patients who restarted medical therapy following the first post‐trial follow‐up, 18 (44%) relapsed following this (*Figure* *2*).

Of the 22 relapses (first or recurrent) during the post‐trial follow‐up, 12 (24%) met more than one criterion for relapse (online supplementary *Figure* *S1* *B*). Twenty‐one (95%) met the LVEF criteria, four (18%) met the NT‐proBNP criteria, and 12 (55%) showed signs and symptoms of HF. Combined with the trial data, in which LVEDVi was also used as a criterion for relapse, 18 (56%) of the 33 patients who met the primary endpoint crossed more than one criteria for relapse (online supplementary *Figure* *S1A*). Twenty‐eight (85%) met the LVEF criterion, 13 (39%) met the criteria for NT‐proBNP, 12 (36%) had signs and symptoms of HF, and 12 (36%) met the LVEDVi criteria.

Of the 22 relapses that occurred during post‐trial follow‐up, seven (32%) were associated with possible precipitating factors, including pneumonia (*n* = 1), poorly controlled hypertension (*n* = 1), pregnancy (*n* = 1), and arrhythmia (*n* = 4; three atrial arrhythmias and one sustained right ventricular outflow tract tachycardia).

### Mortality and morbidity

There were no cardiovascular deaths. One patient died of COVID pneumonitis. Nine (18%) patients had unplanned cardiovascular hospital admissions. Of the admissions, seven (78%) were secondary to arrhythmia (one for sustained right ventricular outflow tract tachycardia, six due to atrial arrhythmia), one (11%) for symptoms of HF during pregnancy, and one (11%) due to pneumonia with co‐existent symptoms of HF. Four (57%) of the seven patients admitted with arrhythmia met the primary endpoint simultaneous with the onset of the arrhythmia. Four (12%) patients had planned cardiovascular visits for ablations and cardioversions, with one patient having two planned visits (one ablation and one cardioversion). Two patients had pre‐existing implantable devices (one implantable cardiac defibrillator [ICD] and one cardiac resynchronization therapy‐defibrillator) both of whom experienced inappropriate shocks due to atrial arrhythmia. Another patient had a loop recorder implanted post‐trial to monitor the burden of AF.

### Changes in medical therapy over time

Of the 13 patients who relapsed for the first time after completion of the trial, two patients did not have therapy withdrawn during the trial, seven had restarted low intensity HF therapy only and four had not restarted medications. A total of 10 (20%) individuals remained on no medications following the initial consult after the trial. One restarted medication at a later stage without subsequent relapse and five patients stayed off medical therapy to the end of follow‐up and did not relapse. Notably, none of these five patients had a history of hypertension, diabetes, left bundle branch block, or a family history of DCM, and two had a history of AF and maintained sinus rhythm following therapy withdrawal (online supplementary *Table* *S2*).

**Figure 3:**
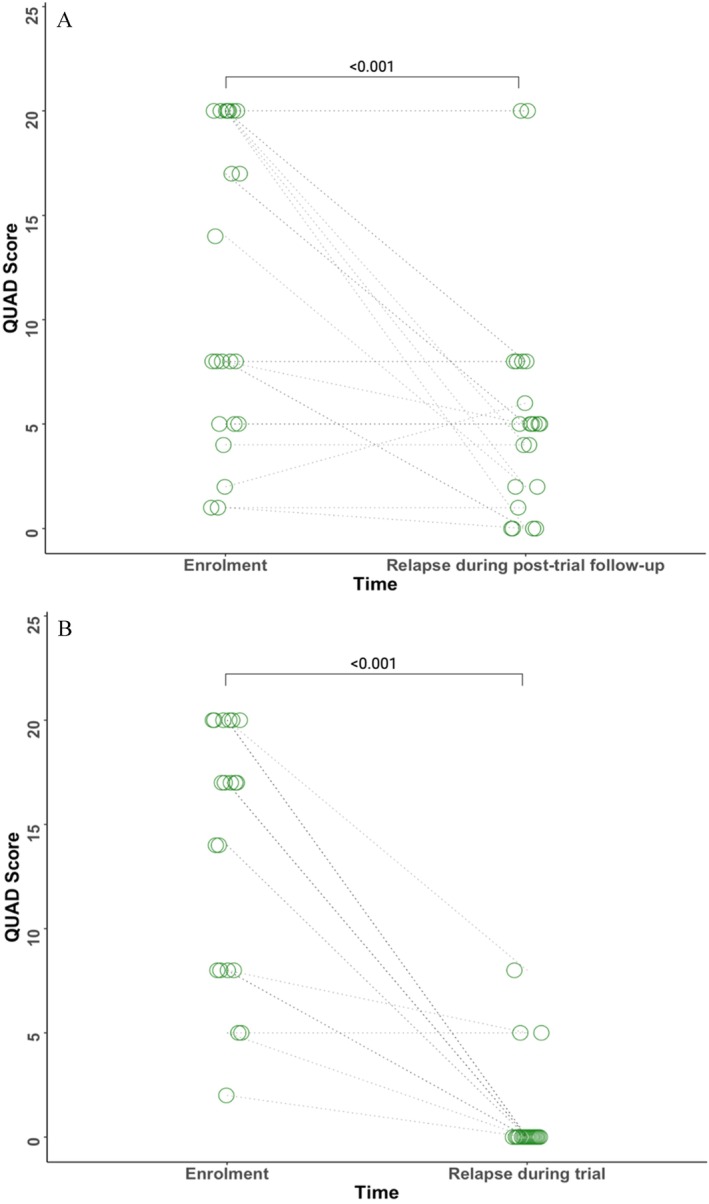
Change in QUAD scores between (*A*) enrolment and relapse during follow‐up (*n* = 22) and (*B*) enrolment and relapse during the trial (*n* = 20). Each circle represents one patient, connected by dotted lines. Brackets represent *p*‐value between two timepoints.

For patients who relapsed during post‐trial follow‐up, the mean QUAD score was lower at the time of relapse compared to enrolment (5 vs. 11, mean difference −6 [−9 to −3]; *p* < 0.001) (*Figure* *3A*). As expected, patients who relapsed during the trial had a lower QUAD score at the point of relapse compared to enrolment (1 vs. 14, mean difference −13 [−16 to −10]; *p* < 0.001) (*Figure* *3B*).

Mean QUAD scores decreased from enrolment to the first post‐trial follow‐up (10 vs. 4), (mean difference −6 [−8 to −4]; *p* < 0.001) (online supplementary *Table* *S3*, *Figure* *S2*). Overall, 38 patients (75%) were re‐established on less intense guideline‐directed medical therapy compared to enrolment. The remaining patients either stayed on medications of the same intensity (*n* = 11, of which 8 relapsed during follow‐up) or higher intensity (*n* = 2, of which 0 relapsed during follow‐up). During follow‐up, there was a significant increase in QUAD score (4 vs. 7, mean difference 3 [1 to 5]; *p* = 0.002) (online supplementary *Table* *S3*, *Figure* *S2*). However, the QUAD score at the end of post‐trial follow‐up remained lower than that at enrolment (12 vs. 8, mean difference −3 [−6 to −1]; *p* = 0.005). Only three patients achieved a maximum QUAD score by the end of the trial. However, this followed a relapse that occurred when the patients were on suboptimal medical therapy (online supplementary *Table* *S4*). For all patients and all drug classes, there was a reduction in prescription from enrolment to first post‐trial follow‐up, followed by a subsequent increase thereafter, but the shifts in prescriptions seem larger for the group that relapsed (online supplementary *Tables* *S5* and *S6*, *Figure* *S2*).

The reasons for therapy intensification included subsequent empirical therapy optimization (39%), deterioration in LVEF (21%), atrial arrhythmia (12%), blood pressure control (10%), signs and symptoms of HF (6%), and non‐sustained ventricular tachycardia on Holter monitoring (1%). Four patients restarted BB therapy only after experiencing an arrhythmia, and another three increased the dose after an arrhythmic event. Meanwhile, half of all increases in ACEi/ARBs were precipitated by hypertension.

### Longitudinal changes in secondary variables over time

**Figure 4:**
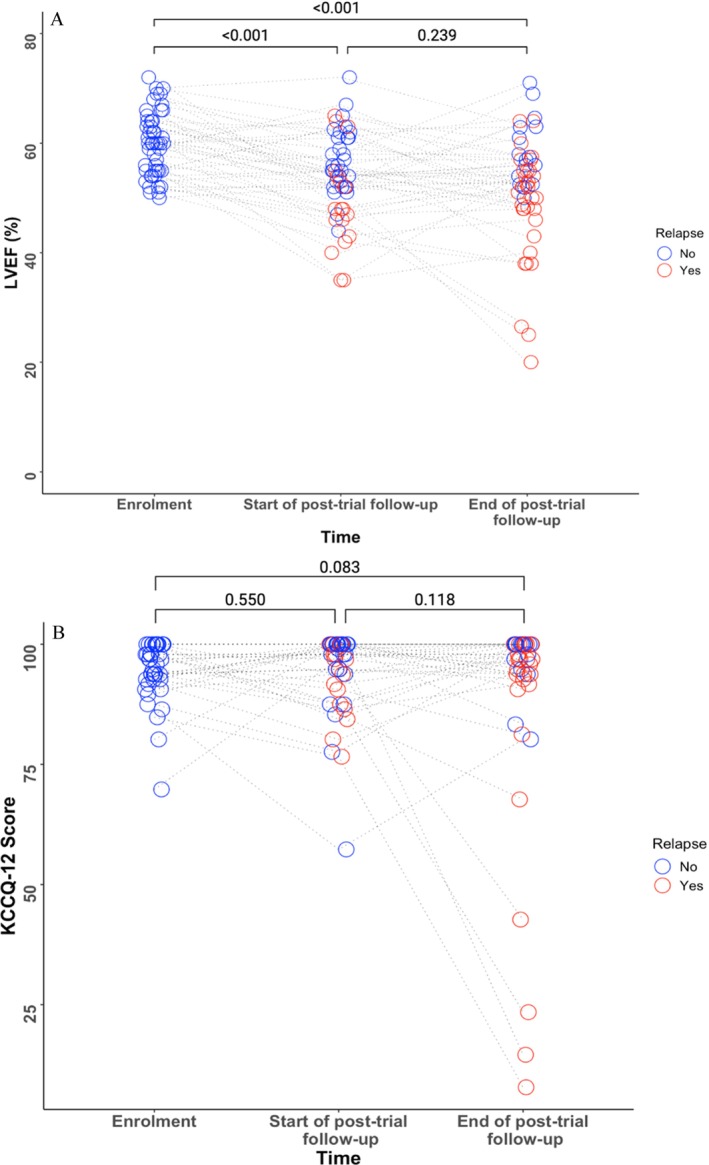
Change in variables (*A*) left ventricular ejection fraction (LVEF) (*n* = 49), (*B*) Kansas City Cardiomyopathy Questionnaire (KCCQ‐12) score (*n* = 40) from enrolment to end of post‐trial follow‐up. Each circle represents one patient, connected by dotted lines. Brackets represent *p*‐value between two timepoints. Red = patients who have relapsed, blue = patients who did not relapse.

There was persistent reduction in mean LVEF from enrolment to the end of the trial (*Figure* *4A*). Mean LVEF was 60% (±6) at enrolment, 54% (±8) at the start of post‐trial follow‐up and 52% (±10) at the end of follow‐up (mean difference between enrolment and end of follow‐up: −8% [−11 to −5]; *p* < 0.001; *Table* *2*). The mean of the lowest recorded LVEF during follow‐up was 46% (±11) (mean difference between enrolment and the lowest recorded during follow‐up: −14% [−18 to −11]; *p* < 0.001; online supplementary *Table* *S3*, *Figure* *S3*). From enrolment to the end of follow‐up, 27 of 51 (53%) patients had an LVEF that decreased by >10% from baseline to <40%.

**Table 2:** Hazard ratios for the primary outcome by patient characteristics for all patients

	Patients	Events	Hazard ratio (95% CI)	*p*‐value
Demographics				
Age (per 10 years)	51	33	1.30 (0.95–1.79)	0.099
Men	34	20	1.0 (ref)	–
Women	17	13	1.74 (0.86–3.51)	0.124
Previous diagnosis and history				
Time since LVEF diagnosis, years	51	33	1.00 (1.00–1.00)	0.277
LVEF at initial diagnosis, %	51	33	0.99 (0.95–1.03)	0.595
Previous AF				
No	39	29	1.0 (ref)	–
Yes	12	4	0.33 (0.12–0.96)	**0.042**
Previous HF admission				
No	19	16	1.0 (ref)	–
Yes	32	17	0.52 (0.26–1.03)	0.062
Causal and other factors of DCM				
Idiopathic	35	22	1.0 (ref)	–
Familial	7	6	1.50 (0.60–3.75)	0.386
Environmental insult	9	6	0.87 (0.33–2.32)	0.783
TTNtv				
No	40	24	1.0 (ref)	–
Yes	11	9	1.57 (0.73–3.39)	0.250
Medications at baseline				
Pre‐trial number of medication(s)	51	33	1.60 (1.05–2.44)	**0.030**
Beta‐blocker				
No	6	4	1.0 (ref)	–
Yes	45	29	1.14 (0.40–3.26)	0.803
MRA				
No	27	14	1.0 (ref)	–
Yes	24	19	2.57 (1.26–5.24)	**0.010**
Loop diuretic				
No	45	28	1.0 (ref)	–
Yes	6	5	1.78 (0.68–4.64)	0.240
QUAD score at pre‐trial baseline	51	33	1.09 (0.99–1.20)	0.069
QUAD score at first post‐trial follow‐up	31	13	1.13 (1.00–1.28)	0.055
Clinical characteristics at enrolment				
HR (per 5 bpm)	51	33	1.06 (0.91–1.23)	0.451
SBP, mmHg	51	33	0.98 (0.95–1.01)	0.123
DBP, mmHg	51	33	0.98 (0.94–1.02)	0.281
LBBB				
No	44	27	1.0 (ref)	–
Yes	7	6	1.46 (0.60–3.55)	0.405
logNT‐proBNP, ng/L	51	33	5.35 (1.49–19.23)	**0.010**
LVEF, %	51	33	0.94 (0.88–1.01)	0.075

Data are *n*, unless otherwise stated. Univariable proportional hazard modelling. Timepoint for characteristics taken from the beginning of TRED‐HF trial until end of post‐trial follow‐up, except for QUAD score post‐trial, which was taken from first follow‐up to end of 5‐year follow‐up.

AF, atrial fibrillation; CI, confidence interval; DBP, diastolic blood pressure; DCM, dilated cardiomyopathy; HF, heart failure; HR, heart rate; LBBB, left bundle branch block; LVEF, Left ventricular ejection fraction; MRA, mineralocorticoid receptor antagonist; NT‐proBNP, N‐terminal pro‐B‐type natriuretic peptide; SBP, systolic blood pressure; TTNtv, titin‐truncating variant.

Mean KCCQ‐12 scores were not significantly different between time points although there was a trend toward a reduction between enrolment and the end of follow‐up (mean difference −7 [−14 to 1]; *p* = 0.083) (*Figure* *4B*, online supplementary *Table* *S3*). Results were qualitatively similar following multiple imputation for the missing data (online supplementary *Table* *S7*). Notably, four patients had scores less than 50 at the end of follow‐up, confirming marked decreases in health status and quality of life. These patients had clinical relapses a year prior, accompanied with worsening signs and symptoms.

Exploratory analyses (*Table* *2*) showed that a history of AF (hazard ratio [HR] 0.33, 95% CI 0.12–0.96; *p* = 0.042), number of medications at baseline (HR 1.60, 95% CI 1.05–2.44; *p* = 0.030), prescription of an MRA at baseline (HR 2.57, 95% CI 1.26–5.24; *p* = 0.010) and baseline log NT‐proBNP (HR 5.35, 95% CI 1.49–19.23; *p* = 0.010) were associated with subsequent relapse at any stage.

## Discussion

Over a median follow‐up of more than 6 years, 65% of participants in the TRED‐HF trial with recovered DCM met the criteria for relapse. The ongoing accumulation of events after the end of the trial, despite most patients restarting some treatment, highlights the longer‐term risk of relapse. Most patients were re‐established on low doses of guideline‐recommended medicines immediately following the trial; titration to target doses often only occurred after evidence of relapse (*Graphical Abstract*). This emphasizes the importance of re‐establishing robust doses of guideline‐directed medical therapy in an expedient fashion if therapy is interrupted for a particular reason, such as pregnancy. Use of low‐dose guideline‐directed therapy appears likely to increase the risk of relapse amongst this heterogeneous population of patients. Patients who went back on the same intensity of therapy as enrolment remained at risk from relapse and LVEF continued to decline during post‐trial follow‐up for the proportion of patients who relapsed during the trial. This raises the possibility of a long‐term legacy effect of withdrawing medications despite their re‐initiation.

Whilst there were no cardiovascular deaths in this cohort, a proportion of patients had unplanned cardiovascular hospitalizations, most commonly due to atrial arrhythmia. This emphasizes the importance of reducing the risk of recurrent atrial arrhythmia, through medical and lifestyle intervention. The two patients who had pre‐existing ICDs both received inappropriate shocks which led to unplanned hospitalizations. Neither experienced an appropriate shock during the follow‐up. Whilst the number of patients with devices is small in this study, similar findings have been confirmed in larger numbers of patients with recovered LVEF.^12^, ^13^ This highlights the importance of weighing up the risks and benefits of continued ICD therapy in the population. Four patients had a marked decline in KCCQ‐12 scores during follow‐up to less than 50, with low scores in all four domains in the questionnaire. This highlights that an important proportion of patients with DCM and HF remission are likely to develop recurrent and debilitating symptoms of HF.

Criteria for relapses were predominantly due to deterioration in LVEF and only about half were accompanied by signs or symptoms of HF. The fact that many patients had a substantial reduction in LVEF on imaging without developing symptoms highlights that many experience silent disease progression, and regular imaging may identify those at risk of recurrent future events. A third of relapses during extended follow‐up had a specific cause including arrhythmia, hypertension, intercurrent infection and pregnancy. This emphasizes the importance of close monitoring during potential stressful events and continued intensification of treatment to enable the vulnerable myocardium to cope with periods of increased workload. BBs may be particularly important in this regard^14^ and may also reduce the risk of atrial and ventricular arrhythmias. Conversely, most patients did not have an identifiable trigger, emphasizing that for now relapse is difficult to predict.

### The possibility of medium‐term recovery of dilated cardiomyopathy and predictors of relapse

Five patients remained off therapy at the end of the extended follow‐up period and remained asymptomatic with normal cardiac function. This suggests that a small proportion of patients might have sustained recovery or even be considered ‘cured’ and may be able to safely stay off therapy long term. At present, there are no reliable indicators of sustained recovery, however, it is interesting to note that around one‐third of patients who remained in remission during follow‐up had a history of AF and had no recurrence. This raises the possibility that the initial presentation was related to AF and that provided rhythm control is maintained, the risk of disease relapse may be reduced. Conversely, however, recurrent or new atrial arrhythmia during follow‐up was common and associated with HF relapse. Other potential predictors of relapse include baseline NT‐proBNP and medication intensity, which were similarly indicators of relapse in the trial data and likely to reflect more severe initial or residual disease. Reliable markers that predict freedom from relapse remain elusive and until these can be identified, continuation of therapy at maximum tolerated doses appears to be the best way to reduce risk.

### Limitations

The benefit of the initial randomization was lost due to the cross‐over design. The study includes a modest number of participants undergoing routine clinical care. Most patients underwent periodic echocardiographic or cardiac magnetic resonance imaging every 12–24 months with measurement of natriuretic peptide levels only if symptoms suspicious of HF developed, as recommended by the research team at the end of the study. It may be argued that a deterioration in LVEF on imaging without symptoms or a change in NT‐proBNP is less clinically important. We cannot exclude the possibility that a small proportion of imaging‐only relapses were due to interobserver error or differences between imaging modalities used during follow‐up. Changes in LVEDVi were not used as a marker of relapse during follow‐up due to the poor agreement between different modalities. Contemporary echocardiography techniques including three‐dimensional measurement of LVEF were typically used. Three‐dimensional echo assessment and cardiac magnetic resonance generally have good agreement for LVEF.^15^ Moreover, a change in LVEF of ≥10% is beyond the expected variability.^16^ It is also possible that some patients might have had undetected relapses if imaging or clinical markers were assessed less frequently, and the true incidence of relapse might therefore be even higher.

## Conclusion

In conclusion, most patients who took part in TRED‐HF relapsed during the trial or in the following 5 years. Complete withdrawal of disease‐modifying HF therapies followed by the re‐initiation of therapy at low doses, triggering events and underlying disease progression are likely to have contributed to the risk of relapse which was associated with unplanned hospitalizations and a deterioration in symptoms in some patients. These results provide further evidence that most patients with remission of DCM remain at risk of relapse and that HF therapy at robust doses should be continued. A relapsing and remitting course is typical for many patients with recovered DCM. Sustained remission without medications may be possible for a small proportion of patients but the default position should be to maintain treatment until the mechanisms and risk of relapse are better understood.

## Supporting information


**Appendix S1.** Supporting Information.
