# Supplementary material for: Long‐term follow‐up of the TRED‐HF trial: Implications for therapy in patients with dilated cardiomyopathy and heart failure remission
Source: Eur J Heart Fail. 2024 Sep 30;27(1):113–23. doi: 10.1002/ejhf.3475 (PMC11798629; doi:10.1002/ejhf.3475)
Supplement: Supplementary file 1 — Appendix S1. Supporting Information. [file EJHF-27-113-s001.docx]

**Appendices**

Long-term follow-up of the TRED-HF trial

| Medication | Target Dose |
| --- | --- |
| ACE-I* | |
| Enalapril | 10–20 mg b.i.d. |
| Lisinopril | 20–35 mg o.d. |
| Ramipril | 5 mg b.i.d. |
| Perindopril | 8 mg o.d. |
| ARNI | |
| Sacubitril/valsartan | 97/103 mg b.i.d. |
| Beta-blockers | |
| Bisoprolol | 10 mg o.d. |
| Carvedilol | 25 mg b.i.d.[e](javascript:;) |
| Metoprolol succinate (CR/XL) | 200 mg o.d. |
| Nebivolol[d](javascript:;) | 10 mg o.d. |
| MRA | |
| Eplerenone | 50 mg o.d. |
| Spironolactone | 50 mg o.d. |
| SGLT2 inhibitor | |
| Dapagliflozin | 10 mg o.d. |
| Empagliflozin | 10 mg o.d. |
| Other agents | |
| Candesartan | 32 mg o.d. |
| Losartan | 150 mg o.d. |

***Appendix Table 1:* Evidence-based doses of guideline-directed medical therapy for heart failure used in calculating QUAD scores, taken from ESC Guidelines (2021).^10^ ***

| ID | Age (enrolment) | Time from diagnosis to enrolment,  yrs | Cause of DCM | TTNtv | Baseline LVEF, % | Baseline  NT-pro-BNP, ng/L | Prior AF | Pre-trial QUAD Score |
| --- | --- | --- | --- | --- | --- | --- | --- | --- |
| 1-003 | 65 | 8.9 | Idiopathic | No | 31 | 87 | Yes | 8 |
| 1-005 | 26 | 5.4 | Idiopathic | Yes | 25 | 62 | No | 5 |
| 1-007 | 50 | 0.8 | Idiopathic | No | 28 | 78 | Yes | 5 |
| 1-018 | 47 | 9.5 | Idiopathic | No | 33 | 65 | No | 8 |
| 1-044 | 22 | 0.5 | Environmental Insult | No | 33 | 5 | No | 2 |

***Appendix Table 2:* Baseline characteristics of patients who stayed off medications and stayed in remission.** DCM= dilated cardiomyopathy. NT-pro-BNP= N-terminal pro-B-type natriuretic peptide.

|  | Patients (n) | Enrolment | Start of post-trial FU | End of post-trial FU | Enrolment vs start of post-trial FU | | Start of post-trial FU vs end of post-trial FU | | Enrolment vs end of post-trial FU | |
| --- | --- | --- | --- | --- | --- | --- | --- | --- | --- | --- |
|  |  |  |  |  | Mean difference (95% CI) | p value | Mean difference (95% CI) | p value | Mean difference (95% CI) | p value |
| QUAD Score | 51 | 10 (±7) | 4 (±5) | 7 (±6) | -6 (-8 to -4) | <0.001 | 3 (1 to 5) | 0.002 | -3 (-6 to -1) | 0.005 |
| LVEF, % | 49 | 60 (±6) | 54 (±8) | 52 (±10) | -6 (-8 to -4) | <0.001 | -2 (-6 to 2) | 0.239 | -8 (-11 to -5) | <0.001 |
| # KCCQ-12 | 40 | 95 (±6) | 94 (±9) | 88 (±23) | -1 (-4 to +2) | 0.550 | -6 (-13 to +2) | 0.118 | -7 (-14 to +1) | 0.083 |
|  | Patients (n) | Enrolment | Start of post-trial FU | Lowest LVEF during FU | Enrolment vs start of post-trial FU | | Start of post-trial FU vs lowest LVEF during FU | | Enrolment vs lowest LVEF during FU | |
|  |  |  |  |  | Mean difference (95% CI) | p value | Mean difference (95% CI) | p value | Mean difference (95% CI) | p value |
| LVEF, % | 49 | 60 (±6) | 54 (±8) | 46 (±11) | -6 (-8 to -4) | <0.001 | -9 (-12 to -5) | <0.001 | -14 (-18 to -11) | <0.001 |

***Appendix Table 3:*** **Change in secondary variables between enrolment, start of post-trial follow-up and end of post-trial follow-up for QUAD score, LVEF and KCCQ. Also compares lowest LVEF during follow-up.** Data are presented using mean difference (95% CI) and p value via paired t-tests. FU= Follow-up, LVEF=Left ventricular ejection fraction. KCCQ=Kansas City Cardiomyopathy Questionnaire.

|  | Patients | Enrolment vs relapse/start of post-trial follow-up | | Relapse/start of post-trial follow-up vs relapse/end of post-trial follow-up | | Enrolment vs relapse/end of post-trial follow-up | |
| --- | --- | --- | --- | --- | --- | --- | --- |
|  |  | Mean difference (95% CI) | p value | Mean difference (95% CI) | p value | Mean difference (95% CI) | p value |
| Change in QUAD Score | 51 | -9 (-11 to -7) | <0.001 | 3 (2 to 4) | <0.001 | -5 (-8 to -3) | <0.001 |

***Appendix Table 4:* Change in QUAD score** **between enrolment, start of post-trial follow-up and end of post-trial follow-up for all 51 patients.** Relapse values are taken if the patients relapsed during the trial or during post-trial follow-up instead of the actual QUAD score at the end of the period. Data are presented using mean difference (95% CI) and p value via paired t-tests.

|  | Enrolment (n=18) | | | Start of post-trial follow-up (n=18) | | | End of post-trial follow-up (n=18) | | |
| --- | --- | --- | --- | --- | --- | --- | --- | --- | --- |
|  | 0 | <50% target dose | ≥50% target dose | 0 | <50% target dose | ≥50% target dose | 0 | <50% target dose | ≥50% target dose |
| RASi | 0 (0) | 7 (39) | 11 (61) | 9 (50) | 6 (33) | 3 (17) | 8 (44) | 4 (22) | 6 (33) |
| BB | 2 (11) | 8 (44) | 8 (44) | 13 (72) | 4 (22) | 1 (6) | 9 (50) | 7 (39) | 2 (11) |
| MRA | 14 (78) | 0 (0) | 4 (22) | 18 (100) | 0 (0) | 0 (0) | 17 (94) | 0 (0) | 1 (6) |
| SGLT2i | - | - | - | - | - | - | 18 (100) | 0 (0) | 0 (0) |
| QUAD Score  (Mean ±SD) | 8 (±7) | | | 2 (±2) | | | 3 (±3) | | |
| Loop Diuretic | 17 (94) | 0 (0) | 1 (6) | 18 (100) | 0 (0) | 0 (0) | 17 (94) | 0 (0) | 1 (6) |

***Appendix Table 5:* QUAD scores and number of patients that were either not on any dose, on <50% or ≥50% target doses of each medication for patients who did not relapse from enrolment to the end of post-trial follow-up (n=18).** Data is represented by n(%). RASi= Renin-angiotensin system inhibitor. BB= Beta blocker. MRA= Mineralocorticoid receptor agonist. SGLT2i= Sodium-glucose co-transporter-2 inhibitor.

|  | Enrolment (n=33) | | | Prior to Relapse in trial (n=20) | | | Prior to relapse in post-trial follow-up (n=22) | | | End of post-trial follow-up (n=33) | | |
| --- | --- | --- | --- | --- | --- | --- | --- | --- | --- | --- | --- | --- |
|  | 0 | <50% target dose | ≥50% target dose | 0 | <50% target dose | ≥50% target dose | 0 | <50% target dose | ≥50% target dose | 0 | <50% target dose | ≥50% target dose |
| RASi | 0 (0) | 11 (33) | 22 (67) | 17 (85) | 2 (10) | 1 (5) | 12 (55) | 6 (27) | 4 (18) | 1 (3) | 9 (27) | 23 70) |
| BB | 4 (12) | 9 (27) | 20 (61) | 17 (85) | 2 (10) | 1 (5) | 7 (32) | 7 (32) | 8 (36) | 0 (0) | 15 (45) | 18 (55) |
| MRA | 15 (45) | 0 (0) | 18 (55) | 20 (100) | 0 (0) | 0 (0) | 19 (86) | 0 (0) | 3 (14) | 20 (61) | 0 (0) | 13 (39) |
| SGLT2i | - | - | - | - | - | - | 22 (100) | 0 (0) | 0 (0) | 25 (76) | 0 (0) | 8 (24) |
| QUAD Score  (Mean ±SD) | 10 (±7) | | | 1 (±2) | | | 5 (±5) | | | 7 (±6) | | |
| Loop Diuretic | 31 (94) | 0 (0) | 2 (6) | 20 (100) | 0 (0) | 0 (0) | 21 (95) | 0 (0) | 1 (5) | 30 (91) | 0 (0) | 3 (9) |

***Appendix Table 6:* QUAD scores and number of patients that were either not on any dose, were on <50% or ≥50% target doses of each medication for patients who relapsed at least once from enrolment to the end of post-trial follow-up (n=33).** Data is represented by n(%). 20 patients relapsed during the trial and 22 patients relapsed during post-trial follow-up, including 9 who had already had a relapse during the trial. RASi= Renin-angiotensin system inhibitor. BB= Beta blocker. MRA= Mineralocorticoid receptor agonist. SGLT2i= Sodium-glucose co-transporter-2 inhibitor.

|  | Enrolment vs start of post-trial follow-up | | Start of post-trial follow-up vs end of post-trial follow-up | | Enrolment vs end of post-trial follow-up | |
| --- | --- | --- | --- | --- | --- | --- |
|  | Mean difference (95% CI) | p value | Mean difference (95% CI) | p value | Mean difference (95% CI) | p value |
| KCCQ-12 Score, 0-100 | -1 (-4 to +1) | 0.261 | -6 (-13 to +2) | 0.128 | -7 (-14 to 0) | 0.061 |

***Appendix Table 7:* KCCQ score at various time points from enrolment to end of post-trial follow-up after using multiple imputation for missing KCCQ measurements.**

A
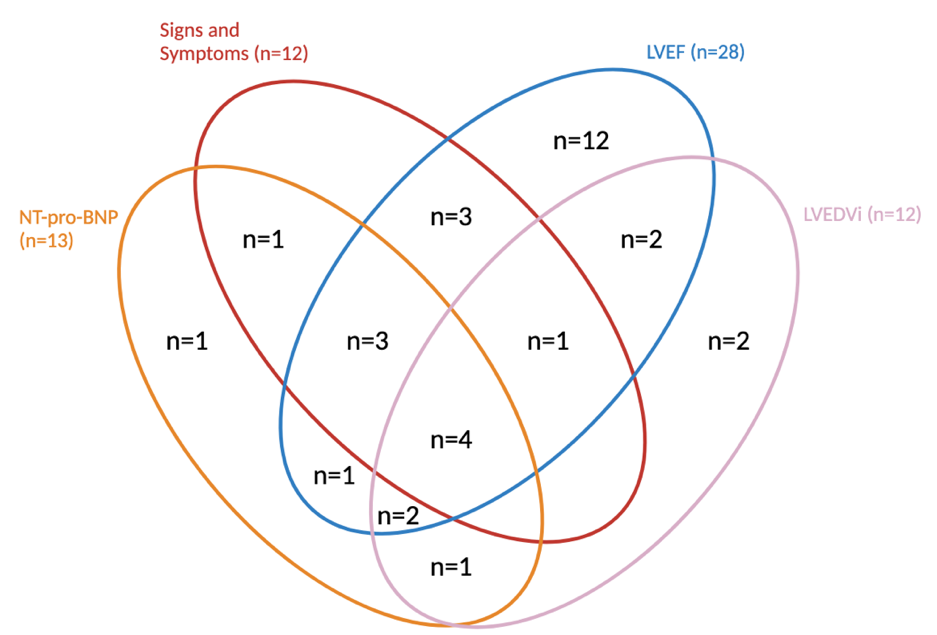


B
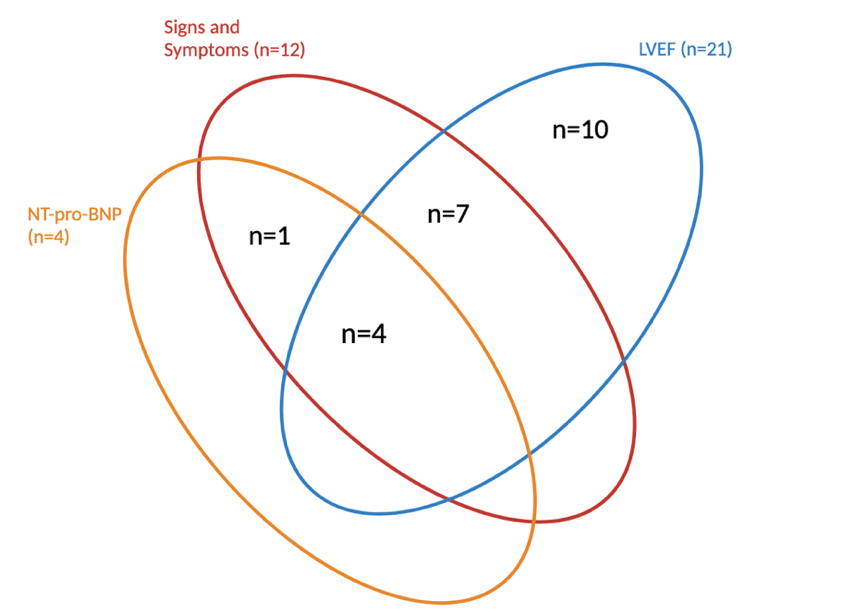


***Appendix Figure 1:* Venn diagrams showing the number of patients meeting certain criteria for primary endpoint from A) enrolment to end of post-trial follow-up and B) start to end of post-trial follow-up.** LVEDVi was not used in the follow-up study as a primary endpoint due to use of different imaging modalities, and hence is not represented in Panel B. NT-pro-BNP=N-terminal pro-B-type natriuretic peptide. LVEF=Left ventricular ejection fraction. LVEDVi=Left ventricular end-diastolic volume index.


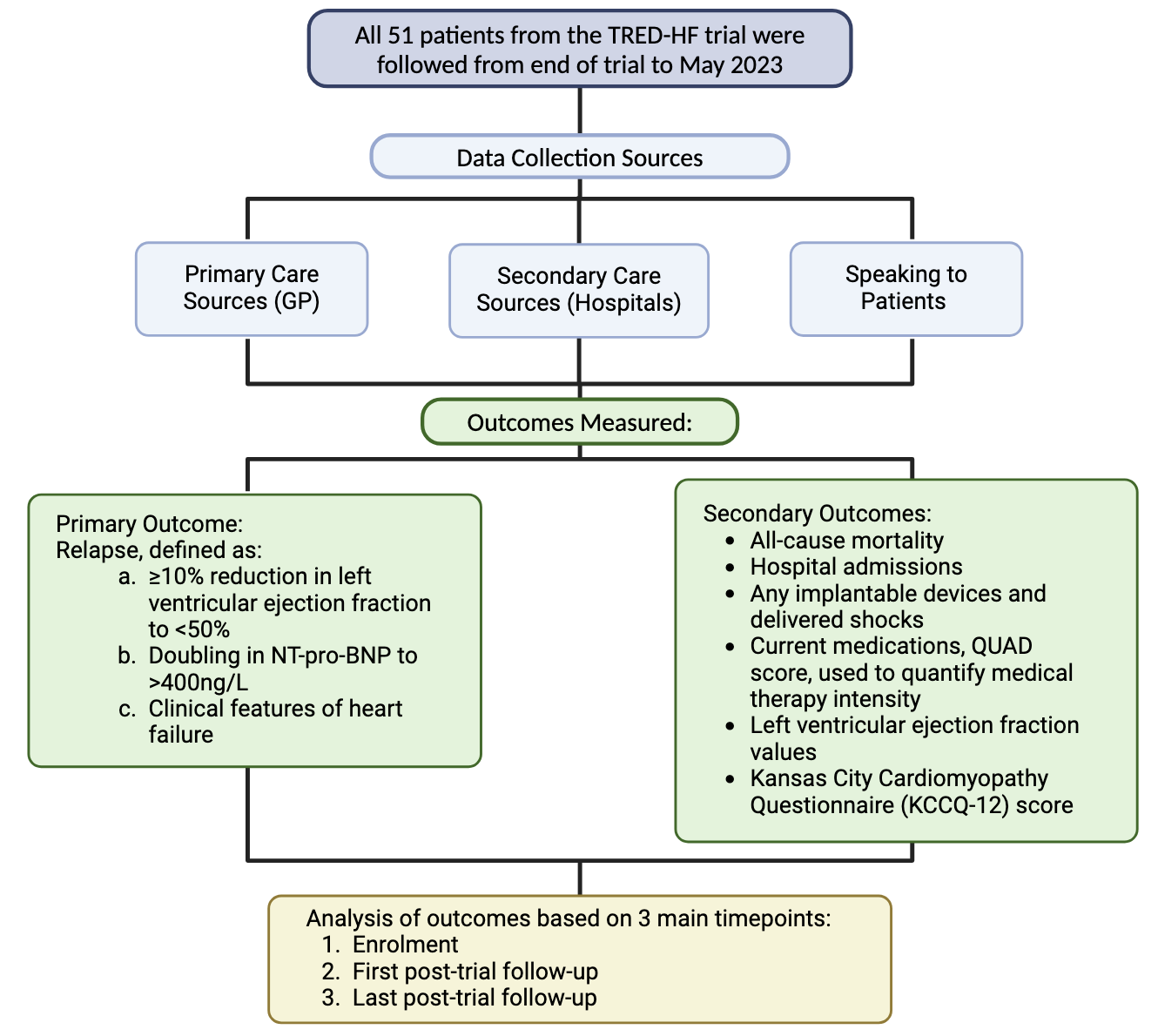


***Appendix Figure 2:*** **Flow chart displaying an overview of the follow-up.** NT-pro-BNP= N-terminal pro-B-type natriuretic peptide.


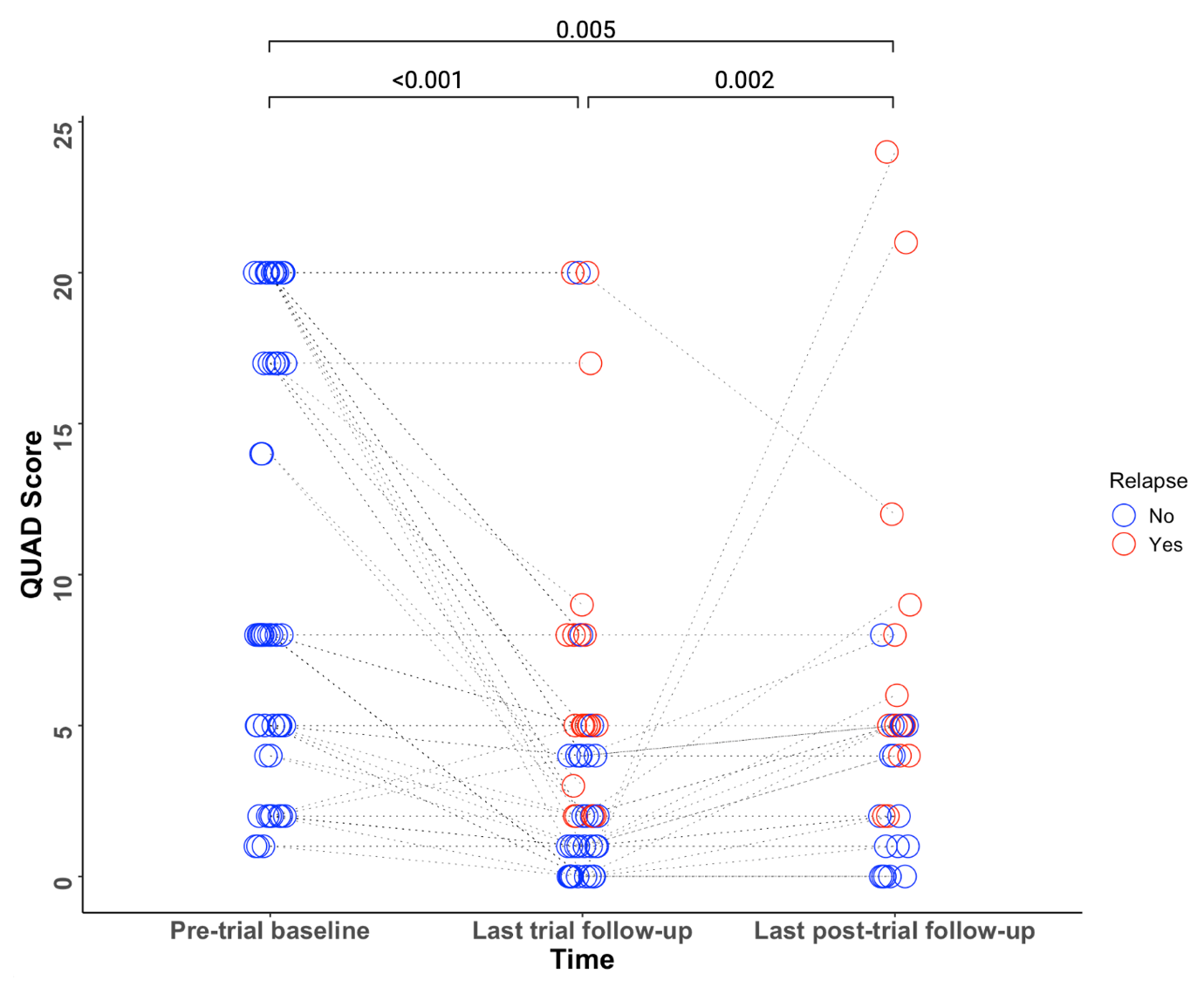


***Appendix Figure 2:* Change in QUAD scores between *A)* Pre-trial baseline and relapse during trial (n=20) and *B)* Pre-trial baseline and relapse during follow-up (n=22).** Mean ±SD are shown in the boxes above for each column. Each circle represents one patient, connected by dotted lines. Brackets represent p-value between two timepoints. Red= patients who have relapsed, blue=patients who did not relapse. Patients who relapsed during the trial were taken out of the plot at “Last post-trial follow-up” for easier comparison but were still included for paired comparisons via paired t-tests.


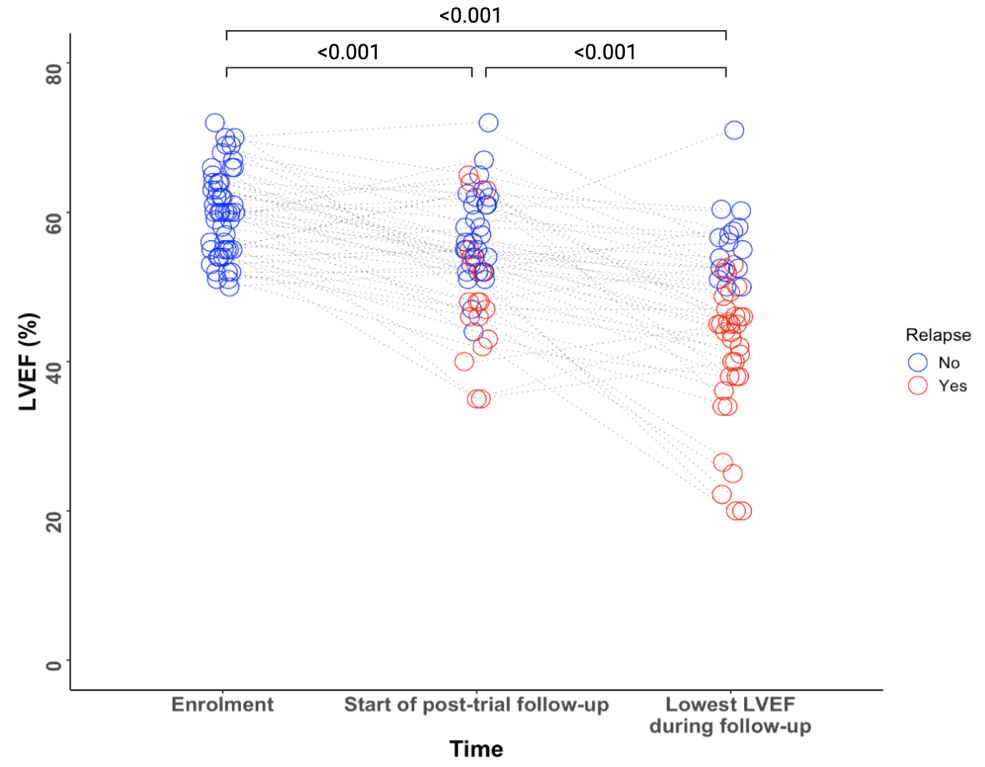


***Appendix Figure 3:* LVEF at enrolment, start of post-trial follow-up and the lowest LVEF during follow-up.** Each circle represents one patient, connected by dotted lines. Brackets represent p-value between two timepoints. Red= patients who have relapsed, blue=patients who did not relapse. LVEF=Left ventricular ejection fraction.
